# A diagnostic dilemma—to operate or not to operate—a rare case report

**DOI:** 10.1093/jscr/rjae300

**Published:** 2024-05-24

**Authors:** Sathish Kumar Thirumurthy, Manjiri Bapat, Ehsan Ahmed

**Affiliations:** Department of General Surgery, NMC Royal Hospital, Al Ghuwair, Sharjah, United Arab Emirates; Department of General Surgery, NMC Royal Hospital, Al Ghuwair, Sharjah, United Arab Emirates; Department of General Surgery, NMC Royal Hospital, Al Ghuwair, Sharjah, United Arab Emirates

**Keywords:** sarcoma, lymphoma, undifferentiated malignancy, plasmacytic infiltrate

## Abstract

A 41-year-old male presented with a swelling in the right flank present since 2 years. Initially, it was small in size but increased in size for the past 6 months. Examination revealed a large swelling in the right flank that was soft in consistency and attached to the deeper muscle. CT scan revealed a heterogenous complex swelling with attachment to the underlying muscle. Core biopsy of the lesion was reported as undifferentiated sarcoma. After immunohistochemistry markers, the diagnosis was revised to a malignancy of a lymphomatous origin. Gene sequencing studies and extensive higher marker studies were done and a final diagnosis of plasmacytic infiltrate of uncertain clinical significance was reported. With no further diagnostic options available, the case still remains to be a diagnostic challenge as the choice of treatment between surgical resection and nonsurgical treatment with chemotherapy and/or radiation cannot be decided upon.

## Introduction

Cases where diagnosis is uncertain pose a challenge to the treating clinician. The choices of treatment between surgical management and nonsurgical management modalities like chemotherapy and radiation are dependent on the type of malignancy. In patients where surgical resection of the malignant lesion is a major procedure with extensive resection and reconstruction, the post treatment quality of life and the possibility of recurrence has to be considered.

## Case report

**Figure 1:**
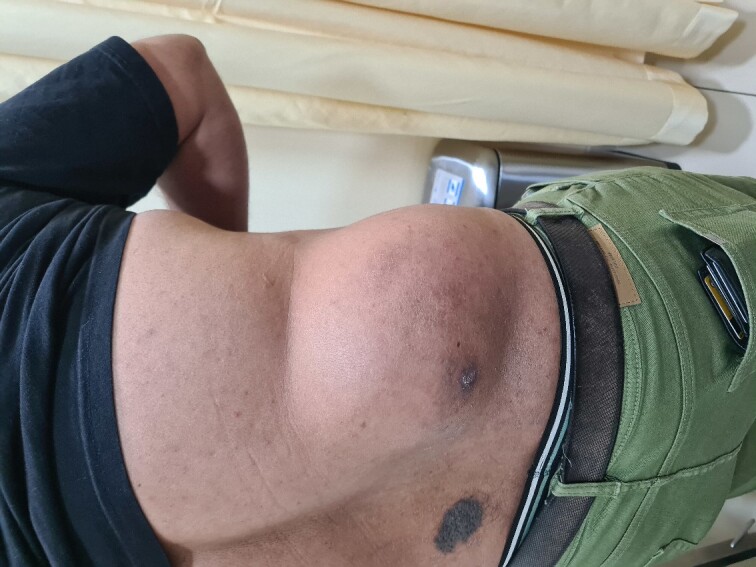
Clinical photograph of the large lesion in the right flank.

**Figure 2:**
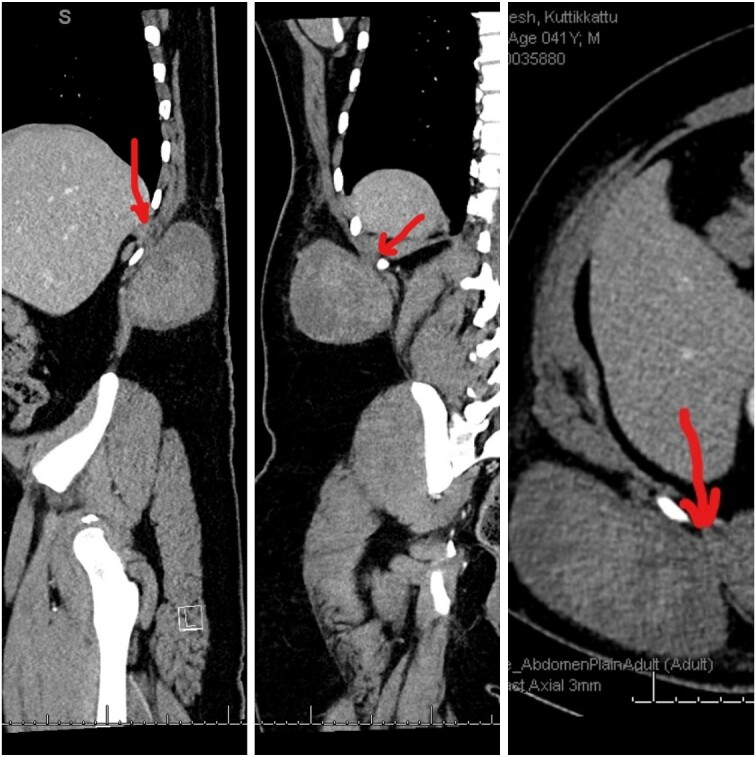
CT images showing the heterogenous lesion in the right flank with the loss of fat plane between the lesion and the underlying muscle at one place.

A 41-year-old male was diagnosed with a right flank swelling that was present for the past 2 years. Initially, the swelling was ~3 cm × 3 cm in size. There was a rapid increase in size since the past 6 months. Clinically, the patient had a right flank swelling measuring 35 cm × 25 cm in size (Fig. 1), which was soft in consistency and attached to the deeper muscular plane. A CT scan was done that revealed the same findings (Fig. 2).

Core biopsy of the lesion was done. Histopathology reported as dedifferentiated liposarcoma/poorly differentiated malignant fibrous histiocytoma [1]. Immunohistochemistry markers were done to evaluate the lesion. Findings were as follows. AE1/AE3 (negative), MYO D1 (negative), Myogenin (negative), A1 Antitrypsin (positive), and A1 Anti chymotrypsin (positive). The histopathology report was revised to undifferentiated malignant fibrous histiocytoma. Further immunohistochemistry markers were done. The findings were as follows: CD 45 (positive), CD 68 (positive in scattered histiocytes), S 100 protein (negative) and Desmin (negative). The histopathology report was then revised to malignant lymphomatous proliferation, most likely Hodgkins Lymphoma. As the diagnosis did not correlate with the clinical findings and as there was disparity in the type of malignancy that was reported, molecular studies were done to arrive at a definitive diagnosis of the lesion. The molecular studies done showed a predominant population of mature plasma cells with low Ki67 proliferation index. Immunoglobin Gene Rearrange V (Negative), MYD88 L265P Gene mutation Analysis (Negative), Ebstein Barr virus encoded RNA (EBER) (Negative), Kappa and Lambda ISH—plasma cells that were heavily skewed toward kappa light chain expression with EBER(negative). The previously done markers CD1a, CD19, CD56, CD86-PGM1, CD117, CD138, CD163, ALK, BRAF V600E, cyclin D1, Factor 13A, HHV8, IgG, IgG4, Langerin, MUM1, OCT2, PAX5 and S100 were repeated. And the findings were same as that of the previous studies.

A final diagnosis of plasmacytic infiltrate, kappa light chain skewed, associated with individually distributed mono nuclear cells of uncertain lineage and of uncertain clinical significance, was reported.

## Discussion

This case has been reported in line with the SCARE criteria [2].

Dedifferentiated liposarcoma and malignant fibrous histiocytoma [3] were initially considered in this patient. Rapidly progressive lympho proliferative lesions in the subcutaneous plane are extremely rare. The lesion has been present for 2 years with a recent rapid increase in size. Clinically and radiologically, it is seen in the subcutaneous plane with no deeper extension except for the attachment to the underlying muscle at one place (Fig. 2). Histopathology findings were that of a fibrous tissue with diffuse proliferation of mature plasma cells with prominent Mott cells. Immunohistochemistry studies were done, which revealed the following: AE1/AE3 (negative), MYO D1 (negative), Myogenin (negative), A1 Antitrypsin (positive) [4], A1 Anti chymotrypsin (positive), CD 45 (strongly positive), CD 68 (positive) [5], S 100 protein (negative) and Desmin (negative). The lesion also had mature plasma cells with a low Ki67 proliferation index. Immunoglobin Gene Rearrange V (Negative), MYD88 L265P Gene mutation (Negative), EBER (Negative), Kappa and Lambda ISH—plasma cells heavily skewed toward kappa light chain expression with EBER (Negative). Other markers like CD1a, CD19, CD56, CD86-PGM1, CD117, CD138, CD163, ALK, BRAF V600E, cyclin D1, Factor 13A, HHV8, IgG, IgG4, Langerin, MUM1, OCT2, PAX5 and S100 were all negative.

Although heavily kappa skewed, the CD 138 and MUM1 plasma cells demonstrated normal co expression of CD19 and CD45. Extremely rare plasma cells expressed CD56 but they did not show aberrant expression of CD117 or cyclin D1 [6]. The plasma cells did not exhibit an increased IgG4:IgG ratio. The individually interspersed large mono nuclear cells expressed only cyclin D1 and were negative for all other antigens. The overall Ki-67 proliferation was very low (<5%) [7]. The staining observed on Alpha-1-anti chymotrypsin and Alpha-1-antitrypsin could be attributed to high background staining.

Additionally, MYD88 L256P [8] abnormality is highly associated (>90%) with the pathological diagnosis of lympho plasmocytic lymphoma [9] and the clinical syndrome of Waldenstrom macroglobulinemia [10]. The MYD88 L256P mutation is also identified in some large B-cell lymphomas [11]. In this case, this mutation was not observed. The plasma cells retained co expression of CD19 and CD45 (which is classically lost in plasmacytomas) [12]. In the absence of a definitive clonal marker, a diagnosis of a plasma cell neoplasm cannot be made as the plasma cells may represent a kappa skewed, but reactive, process. The individually distributed mono nuclear cells associated with the plasmacytic infiltrate only stained for cyclin D1 and did not demonstrate expression of any lineage specific markers (no lymphoid, plasma cell, histiocytic, epithelial, myogenic- or melanoma-specific marker expression) and they were negative for ALK and HHV8 [13]. Given their appearance and association with a brisk plasma cell infiltrate, the possibility of the Rosai−Dorfman disease [14] was considered. But the mononuclear cells were negative for all markers that are aberrantly expressed in this disease. As no lineage could be established for the mononuclear cells, it was impossible to determine their clinical significance.

To proceed with surgery in this case was contemplated [15]. But without a definitive diagnosis, resection of this swelling and reconstruction was a major operation that could not be justified in the absence of a definitive diagnosis.

This case is being studied for its rarity for a massive subcutaneous swelling with features of malignancy and yet being unable to arrive at a definitive diagnosis after a huge array of diagnostic tests that were done. With advanced diagnostic tools available in current clinical practice, such diagnostic dilemmas are very rare to come across in surgical practice.
